# Abnormal Mitochondrial Quality Control in Neurodegenerative Diseases

**DOI:** 10.3389/fncel.2020.00138

**Published:** 2020-06-23

**Authors:** Xu Yan, Biyao Wang, Yue Hu, Sijian Wang, Xinwen Zhang

**Affiliations:** ^1^The VIP Department, School and Hospital of Stomatology, China Medical University, Liaoning Provincial Key Laboratory of Oral Diseases, Shenyang, China; ^2^Center of Implant Dentistry, School and Hospital of Stomatology, China Medical University, Liaoning Provincial Key Laboratory of Oral Diseases, Shenyang, China

**Keywords:** neurodegenerative diseases, mitochondrial quality control, mitochondrial dynamics, mitophagy, mitochondrial biogenesis, inflammation

## Abstract

Neurodegenerative diseases, including Alzheimer’s, Parkinson’s, Huntington’s, and amyotrophic lateral sclerosis, are characterized by a progressive loss of selective neuron subtypes in the central nervous system (CNS). Although various factors account for the initiation and development of these diseases, accumulating evidence shows that impaired mitochondrial function is a prominent and common mechanism. Mitochondria play a critical role in neurons and are involved in energy production, cellular metabolism regulation, intracellular calcium homeostasis, immune responses, and cell fate. Thus, cells in the CNS heavily rely on mitochondrial integrity. Many aspects of mitochondrial dysfunction are manifested in neurodegenerative diseases, including aberrant mitochondrial quality control (mitoQC), mitochondrial-driven inflammation, and bioenergetic defects. Herein, we briefly summarize the molecular basis of mitoQC, including mitochondrial proteostasis, biogenesis, dynamics, and organelle degradation. We also focus on the research, to date, regarding aberrant mitoQC and mitochondrial-driven inflammation in several common neurodegenerative diseases. In addition, we outline novel therapeutic strategies that target aberrant mitoQC in neurodegenerative diseases.

## Introduction

Mitochondria are highly dynamic double-membrane organelles that play a critical role in energy generation, metabolite biosynthesis, intracellular calcium homeostasis, immune responses, and apoptosis (Verdin et al., 2010). Mitochondria generate ATP *via* oxidative phosphorylation (OXPHOS), where electrons are passed from the high-energy substrate to oxygen by means of the electron transport chain (ETC). In addition to ATP production, reactive oxygen species (ROS) can be formed (Sousa et al., 2013), which can damage proteins, membrane lipids, and nucleic acids. The stability of mitochondrial quality and the integrity of mitochondrial morphology are necessary for the normal number, distribution, and correct functions of the organelle.

While mitochondria play a significant role in sustaining cell survival and metabolic homeostasis, mitochondrial dysfunction causes ATP deficiency, an overload of superoxide anions, an increase in proapoptotic molecules, and, ultimately, cell death. Cells have therefore developed a mitochondrial quality control (mitoQC) system to overcome mitochondrial defects, which include mitochondrial proteostasis, mitochondrial biogenesis, mitochondrial dynamics, and mitophagy (Anzell et al., 2018). Mitochondrial quality control thus represents the net balance between the biogenesis rate and degradation rate (Dominy and Puigserver, 2013). Specifically, the homeostasis of mitochondrial dynamics, fission, and fusion maintains the morphology and volume of mitochondria. Mitochondrial biogenetic failure, characterized by cytoplasmic calcium elevation, oxidative stress, and depletion of mitochondrial DNA (mtDNA), is a key stimulator of inflammatory response pathways (Mills et al., 2017). Eliminating dysfunctional mitochondria is achieved *via* various pathways, especially the Parkin/PINK1 pathway (Whitworth and Pallanck, 2017).

In the central nervous system (CNS), mitoQC plays a critical role in neurons as they are long-lasting cells, consequently accumulating defects in themselves (Khalil and Liévens, 2017). As a result of mitochondrial dysfunction, neurons cannot meet the extremely high demand of energy and metabolism, resulting in neuronal degeneration (Meng et al., 2019). Indeed, nonreversible mitochondrial impairment has been shown to trigger cellular damage in neurons, which, in turn, initiates both apoptosis and necrosis (Galluzzi et al., 2009). In addition, defective mitochondria can disrupt glia. It has been reported that mitochondrial fission regulates the activation of nuclear factor κ light-chain enhancer of activated B cells (NF-κB) and mitogen-activated protein kinase (MAPK) signaling and subsequently regulates the release of proinflammatory mediators. Moreover, mitochondrial ROS generation is regulated by mitochondrial fission in microglia (Park et al., 2013, 2015). Chronic activation of microglia may inhibit the expression of proinflammatory mediators, such as cytokines and ROS, contributing to neuronal defection. In addition, the accumulation of mitochondrial-derived ROS has been shown to activate the NLRP3 inflammasome-dependent inflammatory pathway in order to elicit chronic inflammation, because the NLRP3 inflammasome complex triggers the production of interleukin 1β (IL-1β; Zhou et al., 2011). In astrocytes, IL-1β can induce mitochondrial fragmentation and impair the respiration rate, disrupting cellular balance in the CNS (Motori et al., 2013). Therefore, aberrant mitoQC seems to be a central node that mediates the vicious cycle between neuronal and glial injuries.

Herein, we first summarize the molecular basis of mitoQC including mitochondrial biogenesis, proteostasis, dynamics, and mitophagy. We then concentrate on the status of abnormal mitoQC in the initiation and progression of a number of common neurodegenerative diseases.

## Fundamentals of mitoQC

### Mitochondrial Biogenesis

New mitochondria and the proteins involved in OXPHOS are generated *via* mitochondrial biogenesis in order to replace defective mitochondria (Li et al., 2017). Activated biogenesis plays a critical role in preventing cell death, thereby maintaining the energy production and integrity of mitochondria (Khalil and Liévens, 2017).

The interaction between peroxisome proliferator-activated receptor γ coactivator 1α (PGC-1α) and nuclear receptors (e.g., PPAR-γ, estrogen-related receptor α) or transcription factors such as nuclear respiratory factors (NRFs) harmonizes the generation of mitochondrial components between the nuclear and mitochondrial genomes (Puigserver and Spiegelman, 2003). Nuclear respiratory factors modulate the release of respiratory complex subunits (Wu et al., 1999), the proteins involved in mitochondrial import (Blesa et al., 2007) and heme biosynthesis (Braidotti et al., 1993). Furthermore, NRFs modulate the production of mitochondrial transcription factor A (TFAM), which is involved in mtDNA transcriptional and replication processes (Virbasius and Scarpulla, 1994). Hence, activation of the PGC-1α–NRF–TFAM pathway leads to synthesis of both mtDNA and proteins and the generation of new mitochondria.

In addition to PGC-1α, other factors are involved in mitochondrial biogenesis, such as silent mating type information regulation two-1 (SIRT1) and adenosine monophosphate–activated protein kinase (AMPK). Silent mating type information regulation two-1 can directly bind to and activate PGC-1α in order to modulate transcription of respiration-related genes in mitochondria (Lagouge et al., 2006). In addition, during energy-demanding periods, AMPK kinase inhibits ATP metabolism while triggering ATP production. Subsequently, AMPK stimulates mitochondrial biogenesis and activates PGC-1α by direct phosphorylation (Jager et al., 2007) or by indirect stimulation of SIRT1 (Cantó et al., 2009). Therefore, the AMPK–SIRT1–PGC-1α axis plays a pivotal role in stabilizing energy metabolism in defective cells.

### Mitochondrial Proteostasis

In order to deal with mitochondrial stress, there is constant crosstalk between the mitochondria, the nucleus, and the cytosol, triggering transcriptional, translational, and posttranslational programs, aiming at restoring correct mitochondrial function. One major adaptive pathway activated by this mitochondria-nuclear communication is the mitochondrial unfolded protein response (UPR^mt^), which ensures that mitochondrial-specific proteins are correctly translated, folded, and degraded within the organelles in response to stress (Jovaisaite and Auwerx, 2015). Maintaining mitochondrial proteostasis is achieved by chaperones that fold and assemble proteins and proteases that degrade excessively damaged proteins (Bragoszewski et al., 2017). Heat shock proteins (Hsps) import newly generated mitochondrial proteins from the cellular matrix into mitochondria, while maintaining their initial structure (Ostermann et al., 1989; Voos et al., 1996; Liu et al., 2001). Heat shock proteins also retain damaged mitochondrial proteins in order to prevent them from aggregation when they are exposed to mitochondrial stress (Bender et al., 2011). If proteins become irreparable, Hsp70 recognizes and degrades them (Wagner et al., 1994). Mitochondrial proteases function to eliminate damaged or misfolded proteins inside mitochondria. There are four AAA+–ATP-dependent proteases responsible for mitochondrial protein quality control, including the i-AAA protease, the m-AAA protease, LON, and ClpXP (Lebeau et al., 2018), which localize to the inner membrane or the mitochondrial matrix. Irreparable proteins are unfolded through an ATP-dependent process mediated by the AAA+ domains of these proteases (Puchades et al., 2017) and are, subsequently, translocated to a protected proteolytic core for degradation. In addition, the ubiquitin-protease system selectively tags the proteins of the outer mitochondrial membrane (OMM) with a lysine 48-linked polyubiquitin chain, resulting in their elimination (D’Amico et al., 2017).

### Mitochondrial Dynamics: Fission and Fusion

Mitochondrial fission and fusion construct a constant dynamic network, which controls the morphology and population of mitochondria and allows the upgrade of substrates. The dynamic balance is critical for regulating mitochondrial division in cell division and differentiation, as well as for maintaining mitochondrial integrity and cell survival under conditions of stress (Twig et al., 2008). Mitochondrial fission facilitates the segregation of damaged mitochondria from healthy mitochondria, whereas mitochondrial fusion allows two mitochondria to exchange components, such as DNA, proteins, and metabolites, in order to repair each other (Twig et al., 2008; van der Bliek et al., 2013; Pickles et al., 2018). The equilibrium of fission and fusion provides a balance between small defective mitochondria and long interconnected mitochondrial networks, which is crucial for normal mitochondrial function and, accordingly, cell function (Calo et al., 2013).

Mitochondrial fission is mediated by dynamin-related protein 1 (Drp1), a GTPase (Smirnova et al., 2001). Upon activation, Drp1 can relocate to the OMM, where it forms oligomers that can wrap around mitochondria, and eventually divide a mitochondrion into two separate organelles *via* GTP hydrolysis (Smirnova et al., 2001). Drp1 has been shown to interact with four mitochondrial receptor proteins on the OMM, including fission 1 (Fis1), mitochondria fission factor (Mff), mitochondrial dynamics protein of 49 kDa (MID49), and MID51 (Palmer et al., 2011; Zhao et al., 2011). Recent studies have also demonstrated that mitochondria-associated membranes (MAMs) and the endoplasmic reticulum (ER) assist the recruitment of Drp1 to the OMM, which recapitulates the status of mitochondrial division sites (Friedman et al., 2011). While Drp1 governs the process of mitochondrial fission, there is also a small amount of Drp1-independent mitochondrial fission (Roy et al., 2016). The cytoskeleton and its motor proteins can generate a traction force, which is able to tear the mitochondria into two pieces. In mammalian cells, dynein drags mitochondria to move along microtubules, whereas kinesin motors move in the opposite direction. If mitochondria are pulled by both dynein and kinesin, they can be cut into two parts (Schwarz, 2013; Lin and Sheng, 2015). Furthermore, during cell division, the actin-mediated contractile ring may generate the force whereby mitochondria can be torn into two separate structures.

Fusion of the inner mitochondrial membrane (IMM) is regulated by optic atrophy 1 (OPA1), and fusion of the OMM is mediated by mitofusins (Mfns). Long isoforms of OPA1 (L-OPA1) are destined for proteolysis by intramitochondrial proteases and converted into short isoforms called S-OPA1 (Song et al., 2007). It has been reported that L-OPA1 is tagged on the IMM, whereas S-OPA1 is tagged on the intermembrane space (Satoh et al., 2003; Cipolat et al., 2006; Ishihara et al., 2006). Long isoforms of OPA1 interact with S-OPA1 in order to modulate IMM fusion (Song et al., 2007; Zick et al., 2009). However, under stress, the decreased mitochondrial membrane potential (Δψm) induces massive conversion of L-OPA1 to S-OPA1, thus changing the fusion progress (Head et al., 2009). Outer mitochondrial membrane fusion is modulated by Mfn1 and Mfn2, which have very similar sequences (Santel and Fuller, 2001). Mitofusins can interact with each other and form homodimers or heterodimers because Mfns have a GTPase domain and two coiled-coil domains (Rojo et al., 2002; Chen et al., 2003). Mitofusins are activated by GTP hydrolysis and then modulate their structure, causing the fusion of the OMM in surrounding mitochondria (Chen et al., 2003).

### Mitophagy

Mitophagy plays a critical role in the degradation and recycling of defective mitochondria (Soubannier et al., 2012; McLelland et al., 2014) and functions by eliminating damaged mitochondria and inhibiting the production of ROS and apoptotic-related factors. During the process of mitophagy, mitochondria are swallowed by phagophores and then form an autophagosome that subsequently fuses with a lysosome. Mitophagy requires adequate lysosomes, and therefore, mitophagy often takes place in the perinuclear space of cells and axons (Soubannier et al., 2012; McLelland et al., 2014).

Mitophagy has several mechanisms that are predominantly regulated by the PINK1/Parkin pathway (Suen et al., 2010; Rub et al., 2017), which is a pathway that is involved in Parkinson’s disease (PD). Mitophagy involves initially removing fragmented mitochondria with a lower mitochondrial membrane potential (Δψm), whereas those with a high Δψm undergo fusion (Twig et al., 2008). In mitophagy, mitochondrial fragmentation is dominantly induced by the ubiquitination and degradation of mitofusions *via* the PINK1/Parkin pathway (Gegg et al., 2010; Ziviani et al., 2010). Second, immobilized PINK1 and Parkin proteins cause degradation of Miro, which connects mitochondria to microtubule motors and subsequently leads to mitochondrial dysfunction (Kane and Youle, 2011). Blocking of the mitochondria—in this way—leads to their engulfment *via* autophagic phagophores. Third, the aforesaid pathway recruits autophagy initiators, such as Unc51-like kinase l (Lazarou et al., 2015) and Beclin1 (Michiorri et al., 2010), in order to activate the generation of phagophores surrounding the defective mitochondria. The ubiquitinated mitochondria are then recognized by cytoplasmic adaptor proteins of the phagophores, such as optineurin (Wong and Holzbaur, 2014; Heo et al., 2015) and p62/SQSTM1 (Geisler et al., 2010). Adaptors recognize the OMM ubiquitin-binding domain in order to bind ubiquitinated OMM proteins, while recognizing the LC3-interacting region (LIR) domain in order to bind to the autophagosome-associated protein LC3 (microtubule-associated protein 1A/1B–light chain 3). This binding process is activated by phosphorylation *via* the kinase TANK-binding kinase 1 accompanied by its partner optineurin (Matsumoto et al., 2015; Moore and Holzbaur, 2016). As the mitochondrial autophagosomes become fused with lysosomes, their content will be degraded *via* lysosomal hydrolases. Ultimately, mitophagy can also occur independently of the PINK1/Parkin pathway. Recently, some new autophagy receptors of mitochondria have been identified, which can directly interact with LC3, including Bcl-2 and adenovirus E1B19 kDa-interacting protein 3 (BNIP3), Nix/BNIP3L (BNIP3-Like; Novak et al., 2010; Rikka et al., 2011), and FUNDC1 (FUN14 domain-containing protein 1; Liu et al., 2012). In addition to mitophagy receptors, cardiolipin redistribution in mitochondria has been identified as being a novel mitophagy pathway. When the mitochondria are defective, cardiolipin is translocated from the IMM to the OMM, where it fuses with phagophores *via* LC3 (Chu et al., 2013).

## Abnormal mitoQC in Neurodegenerative Diseases

**Figure 1:**
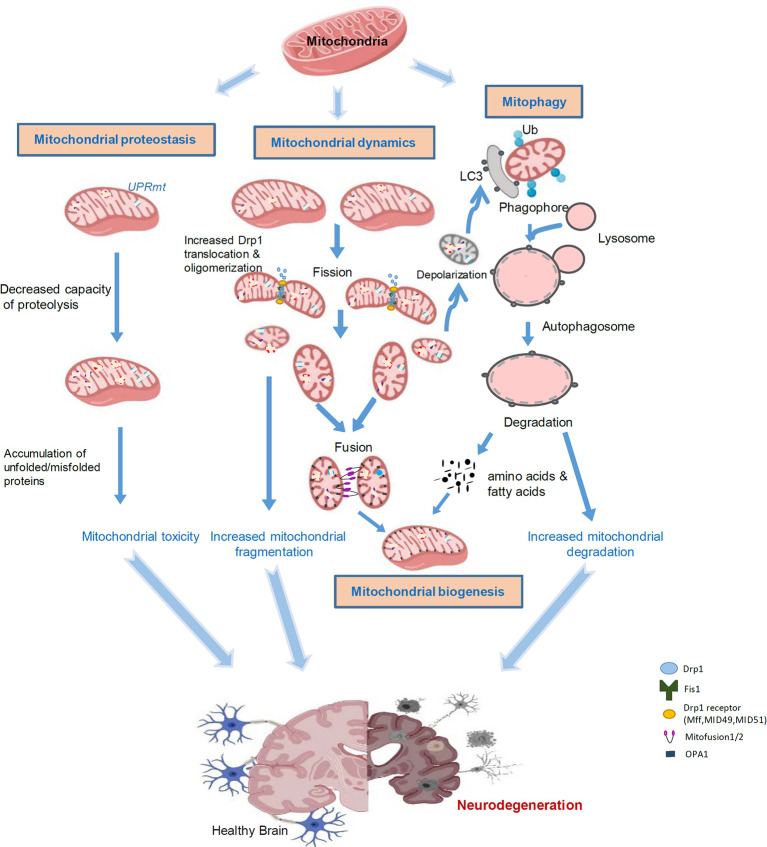
Aberrant mitochondrial quality control leads to neurodegeneration. Mitochondria are highly sensitive to stress and diseases. Accumulation of misfolded proteins within mitochondria induces the UPR^mt^, leading to toxicity in the mitochondria. Mitochondrial stress can cause alterations in mitochondrial dynamics. An increase in Drp1-mediated fission of mitochondria leads to mitochondrial fragmentation and subsequent mitochondrial damage. Certain impaired mitochondria can fuse with other healthy mitochondria in an attempt to salvage that mitochondrion; however, typically, dysfunctional mitochondria will undergo mitophagy. Fragmented and damaged mitochondria can be eliminated by mitochondria-associated autophagy, termed mitophagy. Once the mitochondria are degraded, the cell will recycle the amino acids and fatty acids to enable the remaining healthy mitochondrial network to grow and divide through biogenesis. Aberrant mitophagy results in either an accumulation of damaged mitochondria or excessive degradation of healthy mitochondria. These processes are interconnected in response to stress and collectively lead to neurodegeneration.

Neurodegenerative diseases are characterized with progressive loss of selective neuron subtypes in the CNS; examples of these diseases include Alzheimer’s disease (AD), PD, Huntington’s disease (HD), and amyotrophic lateral sclerosis (ALS). Despite great progress having been made to clarify the pathogenesis of these neurological disorders, the underlying mechanisms are still elusive, and effective therapeutic strategies for these diseases have not yet emerged. Increasing evidence has demonstrated the significant status of mitochondrial abnormality in the pathological states of these neurodegenerative diseases (AD, PD, HD, and ALS). These abnormalities include, but are not limited to, defective mitochondrial morphology/structure, aberrant mitophagy, and impairment of mitochondrial biogenesis, all of which are governed by mitoQC (Figure 1). Furthermore, recent studies have highlighted a central role of mitochondria in innate and adaptive immune responses in CNS cells, linking mitochondrial dysfunction and neuroinflammation. Here, we will discuss previous findings on aberrant mitoQC and mitochondrial-driven inflammation in neurodegenerative diseases, focusing on AD, PD, HD, and ALS.

### Alzheimer’s Disease

Alzheimer’s disease, including familial AD (FAD) and sporadic AD, is an age-related neurodegenerative disease that is characterized by progressive cognitive impairment, memory loss, and mobility disorder. The pathogenesis of AD is complex, involving abnormal metabolism of amyloid β (Aβ), which leads to the deposition of Aβ plaques, hyperphosphorylation of Tau protein, deposition of neurofibrillary tangles, changes of reactive glial cells, and other pathological phenomena (Wang et al., 2017a).

The disequilibrium between generating and clearance of Aβ leads to its accumulation, which initiates the pathogenic cascade that eventually leads to AD (Selkoe and Hardy, 2016). Amyloid β is generated by continuous proteolysis of amyloid precursor protein (APP), mediated by β-secretase and γ-secretase. Presenilin genes 1 and 2 (PSEN1, PSEN2) are necessary for forming γ-secretase complexes to cleave APP and drive Aβ formation (Wang et al., 2017a). Microtubule-associated protein Tau is another major molecule found in AD. As part of the cytoskeleton, microtubules are carriers of various organelles including mitochondria and lysosomes (Buée et al., 2000). Systemic cerebral perfusion of rotenone (an inhibitor of ETC complex I) leads to Tau hyperphosphorylation in neurons, astrocytes, and oligodendrocytes of rats (Hoglinger et al., 2005), suggesting that mitochondrial dysfunction contributes to taupathy. As a major apolipoprotein in the CNS, apolipoprotein E (ApoE) binds the extracellular region of triggering receptor expressed on myeloid cells 2 (TREM2; Atagi et al., 2015), which is expressed on cells of the myeloid lineage, such as microglia and osteoclasts (Colonna and Wang, 2016). Soluble TREM2 enhances the proliferation and migration of microglia, gathering around amyloid plaques, as well as the ingestion and disintegration of Aβ (Zhong et al., 2019). In defective TREM2, there is an increase in the deposition of amyloid plaques and a decrease in the clustering of the microglia around newly formed amyloid plaques, and levels of the plaque-associated ApoE are reduced (Parhizkar et al., 2019).

#### Abnormal Mitochondrial Dynamics and AD

Fragmented mitochondria and abnormal mitochondrial distribution and trafficking in neurons have been observed in numerous AD models in cell culture and animals, as well as in AD patient postmortem brains (Wang et al., 2017b, 2009; Pérez and Quintanilla, 2017). The mitochondrial fission and fusion proteins are differently altered in the AD hippocampus with an upregulation of the mitochondrial fission protein Fis1, accompanied by a decrease in fission protein Drp1, as well as fusion proteins Mfn1, Mfn2, and OPA1 (Wang et al., 2009). Moreover, phosphorylation of Drp1 at Ser616 and S-nitrosylation of Drp1 were shown to be higher in AD patient brains than in control subjects (Wang et al., 2009), confirming the induction of excessive mitochondrial fission. Dynamin-related protein 1 has been reported to interact with Aβ and phosphorylated Tau in AD patient postmortem brains (Manczak et al., 2011; Manczak and Reddy, 2012). Kim et al. (2017) recently reported a significant relevance between specific polymorphisms in the MFN2 gene and AD, suggesting a relationship between genetic polymorphisms during fusion and AD pathogenesis.

Treatment with mitochondrial division inhibitor (Mdivi-1), a putative Drp1 inhibitor, restored Aβ-mediated mitochondrial dysfunctions and neuron synaptic depression, as well as reducing Aβ deposition and memory deficits in the brains of APP/PS1 and CRND8 mice (Baek et al., 2017; Wang et al., 2017b). Similarly, inhibition of Drp1 hyperactivation by P110, a selective Drp1 peptide inhibitor (Qi et al., 2013), reduced the number of fragmented mitochondria and attenuated AD pathology and behavioral deficits in 5XFAD AD mice (Joshi et al., 2018). Dysregulated mitochondrial permeability transition pore (MtPTP) is associated with the metabolic stress observed in AD (Pérez and Quintanilla, 2017). Altering mitochondrial dynamics by downregulation of OPA1 resulted in mitochondrial enlargement and decreased MtPTP function (Piquereau et al., 2012). Similarly, upregulation of Mfn2 and enhancement of mitochondrial fusion reduced the sensitivity of MtPTP, eliminating ROS release (Neuspiel et al., 2005). The mitochondrial protein, Miro, mediates mitochondrial trafficking along the axon (Wang and Schwarz, 2009). The heterozygous Miro mutation [miro(Sd32)] has been previously related to the disposition of Aβ42 in the *Drosophila* model (Iijima-Ando et al., 2009). Inhibition of Miro activated the PAR-1/MARK family kinases, subsequently promoting the pathological phosphorylation of Tau. It should be noted that late-onset neurodegeneration in *Drosophila* could be suppressed by the single knockdown of PAR-1 or Tau (Iijima-Ando et al., 2012). Thus, an imbalance between fusion and fission contributes to AD pathogenesis, and targeting abnormal mitochondrial dynamics could provide therapeutic options for AD.

#### Abnormal Mitophagy and AD

Abnormal accumulation of autophagy vacuoles in neurons is a prominent feature of AD and is likely to be the result of lysosomal dysfunction (increased pH) or secondary to neuronal Ca^2+^ homeostasis disorder (Nixon, 2013). During mitophagy, mitochondria are swallowed by a structure called a phagophore, forming an autophagosome that subsequently fuses with a lysosome, indicating a significant role for lysosomes in mitophagy (Soubannier et al., 2012; McLelland et al., 2014). In FAD patients, mutation of PSEN1 leads to abnormal acidification of lysosome and reduces lysosomal hydrolase activity (Coffey et al., 2014). In both mutant hAPPTg neurons and AD patient brains, Parkin-mediated mitophagy was induced (Ye et al., 2015). As the disease progresses, the cytosolic Parkin levels significantly decreased in the AD patient brain, and mitophagy became inefficient (Ye et al., 2015; Cai and Tammineni, 2016). Furthermore, FAD PSEN1 mutations aggravated lysosomal alkalization, thereby reducing lysosomal hydrolytic activity. This suggests that impaired lysosomal function is a stimulating factor in AD (Coffey et al., 2014). Sirtuin 3 (Sirt3) mediates mitochondrial biogenesis and oxidative challenge, and Sirt3 activates FOXO3 to induce p62 (a major mitophagy protein), which accumulates on ubiquitinated mitochondrial substrates and forms autophagy lysosomes. Sirt3 levels decreased in neurons of APP/PS1 double-mutant AD mice (Tseng et al., 2013), again supporting defective mitophagy in AD. In mouse neuroblastoma cells, Cummins et al. (2019) showed that Parkin translocation to the damaged mitochondria can be reduced by human wild-type (WT; hTau) expression, as well as mutant tau (hP301L). A reduction in Parkin translocation resulted from an abnormal interaction of the projection domain of Tau with Parkin, and thus Parkin is sequestered in the cytosol (Cummins et al., 2019). An experiment based on a rat pheochromocytoma cell line from an AD cellular model showed that resveratrol can inhibit Aβ42-induced mitochondrial damage and enhance mitophagy, whereas 3-methyladenine can inhibit mitophagy mediated by resveratrol and promote apoptosis (Wang et al., 2018a). Cordero et al. (2018) indicated that oleuropein aglycone in extra virgin olive oil can facilitate mitophagy, reducing aggregated proteins and thereby delaying the course of AD. In addition, a study in hippocampal neural stem cells (PS1 M146L NSCs) of AD mice demonstrated that bexarotene, a mitophagy-stimulating drug can alleviate the abnormity of mitochondria (Martín-Maestro et al., 2019). Therefore, aberrant accumulation of dysfunctional mitochondria in AD-affected neurons is likely attributable to an inadequate mitophagy capacity in eliminating increased numbers of damaged mitochondria, and the improvement of mitophagy may be a new starting point for treating AD.

#### Defective OXPHOS, Oxidative Stress, and AD

As the main source of energy supply, mitochondria maintain the integrity and reactivity of neurons (Sheng and Cai, 2012). The activities of complex I (NADH dehydrogenase) and IV (cytochrome C oxidase) have been reported to be significantly reduced in AD (Holper et al., 2019), resulting in defective OXPHOS. The decrease of energy metabolism due to defective OXPHOS causes excessive accumulation of ROS and elevated oxidative stress, which creates a transient feedback loop that further leads to mitochondrial damage (Wang et al., 2005; Sheng et al., 2012). Evidence indicates that CNS cells are more sensitive to Aβ-induced cytotoxicity by promoting ETC activity, mitochondrial OXPHOS, and ROS levels while repressing aerobic glycolysis (Lone et al., 2018). However, in human SH-SY5Y neuroblastoma cells, a higher level of endogenous Aβ was shown to cause no OXPHOS disturbance (Lopez Sanchez et al., 2017), suggesting that APP may downregulate mitochondrial OXPHOS independently. The interaction between AD and mitochondrial OXPHOS remains to be confirmed.

#### Mitochondrial-Driven Inflammation and AD

There are several reports that suggest that at least one type of damage-associated molecular pattern molecule, which stem from mitochondria, can lead to an inflammation response in neuronal and microglial cell lines (Wilkins et al., 2015, 2017). In a mouse microglial cell line, treatment with mitochondrial lysates induced the expression of messenger RNA (mRNA) encoding for tumor necrosis factor α (TNF-α) and IL-8, as well as matrix metalloproteinase 8 (Wilkins et al., 2015). Activated microglia act as innate immune cells in the CNS (Kettenmann et al., 2011), having dual influence on AD progression that depends on the period of activation. As a self-defense mechanism, activated microglia reduces the subtle accumulation of Aβ by stimulating phagocytosis, clearance, and degradation so that it can initiate the process of tissue repairing and wound healing to maintain brain health (Graeber et al., 2011). However, chronic microglial activation leads to the secretion of proinflammatory cytokines, such as IL-1β, TNF-α, and interferon γ (IFN-γ), which induces inflammation, and leads to further neuronal dysfunction (Jiang et al., 2012). The activation of downstream signaling pathways regulates the activities of transcription factors such as NF-κB, leading to oxidative stress and Aβ-induced neuronal cell death (Song et al., 2004; Chongthammakun et al., 2009). Mitochondrial-derived H_2_O_2_ can upregulate or downregulate NF-κB (Takada et al., 2003; Csiszar et al., 2008). Excessive levels of H_2_O_2_ inactivate NF-κB *via* oxidation of its p50 subunit, whereas moderate H_2_O_2_ levels result in IKK- or Syk-induced phosphorylation of IκB, activating NF-κB (Takada et al., 2003; Patten et al., 2010). Proinflammatory neurotoxins have been shown to cause downregulation of TREM2 and vitiation of the phagocytosis of extracellular debris by microglial cells, which alter the microglia phenotype into a neurodegenerative pattern (Deczkowska et al., 2018). Therefore, a vicious cycle between mitochondrial dysfunction and activated microglia-related neuroinflammation could promote the progression of AD. Valproic acid has been shown to prevent cytotoxicity stimulated by Aβ_25-35_ (a fragment of Aβ protein), which subsequently inhibits the activation of NF-κB signaling, decreases the expression of proinflammatory cytokines, and reduces mitochondrial dysfunction in rat PC12 cells (Zhao et al., 2018). In mouse neuroblastoma N2a cells, Zhang et al. (2017) reported that PGC-1α inhibited neuroinflammation and Aβ-induced cell death, which was regulated by the NF-κB pathway. Thus, a therapeutic strategy for AD that targets neuroinflammation, mitochondrial dysfunction, and NF-κB pathway could be feasible.

### Parkinson’s Disease

Parkinson’s disease is the second most common neurodegenerative disease and is characterized by the loss of dopaminergic (DA) neurons in the substantia nigra (SN) area and the formation of cytoplasmic inclusion bodies containing a-synuclein [Lewy body (LB)]. Patients with PD are subject to the degradation of certain behaviors, such as bradykinesia, stiffness, tremors, and gait disorder (Gao et al., 2018). The pathogenesis of PD is complicated and considerable, whereas mitochondrial dysfunction has been increasingly appreciated to be a risk factor of DA neuronal susceptibility (Dauer and Przedborski, 2003; Dawson and Dawson, 2003; Ellis et al., 2005; Yao and Wood, 2009). Neurotoxins, such as 1-methyl-4-phenyl-1,2,3,6-tetrahydropyridine (MPTP) and rotenone, can inhibit complex I to impair respiratory chain function in *in vitro* and *in vivo* models of PD (Langston et al., 1983; Betarbet et al., 2000; Smeyne and Jackson-Lewis, 2005; Testa et al., 2005). Parkinson’s disease susceptible genes, which encode for proteins such as leucine-rich repeat kinase 2 (LRRK2), Parkin, PINK1, and DJ-1, are localized on the mitochondria and interfere with the function of mitochondria (Klein and Westenberger, 2012; Karimi-Moghadam et al., 2018).

#### Abnormal Mitochondrial Dynamics and PD

The protein LRRK2 is encoded by the PARK8 locus, which belongs to the Roc GTPase family, containing a conserved serine-threonine kinase MAPK kinase kinase (MAPKKK) domain (Paisán-Ruíz et al., 2004; Zimprich et al., 2004; West et al., 2007). Mutation of G2019S (Gly2019 to Ser) that occurs in the MAPKKK domain is the primary cause of familial PD and approximately 2% of sporadic PD cases (Cookson, 2010). The kinase activity of LRRK2 is augmented *via* the mutant of G2019S, which has been shown to be correlated with the toxicity of DA neurons (West et al., 2005). The LRRK2 G2019S mutant increases the recruitment of Drp1 to mitochondria (Wang et al., 2012) and phosphorylates Drp1 at Thr595, leading to Drp1-dependent mitochondrial fragmentation and subsequent excessive mitophagy (Su and Qi, 2013). Disruption of mitochondrial fission leads to synaptic loss and DA neuronal cell death (Berthet et al., 2014). Pharmacological and genetic inhibition of Drp1 hyperactivation has been demonstrated to protect DA neurons in an MPTP-induced PD mouse model, and in induced pluripotent stem cells (iPS) cells derived from patients carrying either the LRRK2 G2019S or the α-synuclein (α-syn) mutant (Su and Qi, 2013; Rappold et al., 2014; Filichia et al., 2016; Bido et al., 2017; Seshadri and Alladi, 2019; Geng et al., 2019; Park et al., 2019), by improving both mitochondrial morphology and autophagic flux. PINK1 and Parkin are also crucial for maintaining mitochondrial fusion and fission by mediating the ubiquitination of Mfns (Ziviani et al., 2010). α-Synuclein is a major component of LBs, and its oligomeric forms have been demonstrated to trigger neurotoxicity in PD (Selkoe et al., 2014; Peelaerts et al., 2015). α-Synuclein is widely distributed in the mitochondria and mitochondria-associated ER membrane (MAM; Devi et al., 2008; Guardia-Laguarta et al., 2014). While WT α-syn and its missense mutant, A53T (Lee and Trojanowski, 2006), have been reported to induce mitochondrial fragmentation in a Drp-1-dependent manner, overexpression of the fusion protein OPA1 has been shown to prevent both mitochondrial fragmentation and neuronal cytotoxicity (Martinez et al., 2018). In rat brains overexpressing human A53T–α-syn, the fragmentation of mitochondria and aggregates of α-syn are reduced, and the motor function is normalized by treatment with Mdivi-1 (Bido et al., 2017), further supporting the notion that correcting aberrant mitochondrial dynamics attenuates PD pathology.

#### Abnormal Mitophagy and PD

Depolarized mitochondria cause an accumulation of PINK1 on the OMM. Parkin recruitment to the mitochondria depends on the presence of catalytically active PINK1 (Geisler et al., 2010; Matsuda et al., 2010; Narendra et al., 2010; Vives-Bauza et al., 2010). The recessive mutation of Parkin/PINK1 observed in PD patients blocks the process of mitophagy, which aggravates the morphological destruction of mitochondria and accumulation of the dysfunctional organelle (Ziviani et al., 2010). PINK1 and Parkin have been shown to coordinate the selective clearance of damaged mitochondria *via* mitophagy (Matsuda et al., 2010; Vives-Bauza et al., 2010), and in recent years, there has been a large of amount of literature highlighting the mechanistic link between mitophagy deficiency and PD pathogenesis using cell culture models (Narendra and Youle, 2011; Youle and Narendra, 2011; Pickrell and Youle, 2015; Mouton-Liger et al., 2017; Truban et al., 2017; Miller and Muqit, 2019). Because of the lack of *in vivo* phenotypes in PINK1 and Parkin mutant mice, it is hard to evaluate the impact of PINK1/Parkin-mediated mitophagy deficiency on PD pathology *in vivo* (Goldberg et al., 2003; Perez and Palmiter, 2005; Kitada et al., 2009). Recently, Seibler et al. (2011) reported that neurons derived from iPS cells of PD patients carrying PINK1 missense mutations showed an impairment of Parkin recruitment to mitochondria and increased mitochondrial copy number. In addition, DA neurons derived from iPS cells of patients with Parkin mutation exhibited shortening neurites and sensitivity to stressors (Ren et al., 2015). These findings from the human neuronal model may provide distinct phenotypes that could be amenable to further mechanistic studies in the context of human PD. Dynamin-related protein 1 was recruited from the cytoplasm to the dysfunctional mitochondria together with Ca^2+^ and Zn^2+^ signals, thus causing mitochondrial fragmentation (Abuarab et al., 2017). The fragmented mitochondria may facilitate their engulfment by autophagosomes (Buhlman et al., 2014). It will be interesting to determine the detailed molecular pathway by which Drp1-mediated mitochondrial fragmentation orchestrates the PINK1/Parkin-associated mitophagy.

#### Defective OXPHOS, Oxidative Stress, and PD

In terms of the mtDNA cluster JTWIX, mtDNA-encoded complex I genes are immoderately altered, which has been shown to be strongly related to an increased risk for PD (Autere et al., 2004). These mutations probably alter the efficiency of OXPHOS and result in mitochondrial dysfunction and oxidative stress in PD. Dopaminergic neurons contain high levels of pro-oxidant iron, and low levels of glutathione seem to be the crucial antioxidant in the SN. Transition metals can reduce oxygen to promote ROS production (Wei et al., 2019), so that neurons are especially vulnerable to oxidative stress, which can induce mtDNA mutations. In fact, SN neurons with defective OXPHOS enzymes have high levels of mtDNA mutations in aged human and PD patients (Kraytsberg et al., 2006). The reduction of membrane potential, overloaded levels of Ca^2+^ (Gandhi et al., 2009), and increased production of ROS in mitochondria have been observed in PINK1 knockout fibroblasts and neurons (Heeman et al., 2011). The mutation of DJ-1 leads to misfolded α-syn aggregates in DA neurons of PD patients (Zondler et al., 2014); the lack of DJ-1 decreases the consumption of hydrogen peroxide and the clearance of ROS in the brain mitochondria, as well as increasing oxidative stress levels, leading to the death of DA neurons (Lopert and Patel, 2014).

#### Mitochondrial-Driven Inflammation and PD

PINK1 and Parkin play a crucial role in adaptive immunity by suppressing the presentation of mitochondrial antigens (Matheoud et al., 2016), which suggests an involvement of autoimmune mechanisms in PD etiology. Mitochondria isolated from the brains of PINK1 knockout mice exhibit increased vulnerability to oxidative stress caused by inflammation (Akundi et al., 2011). Neurons derived from iPS cells of patients with LRRK2 mutations have shown impaired NF-κB signal transduction and a reduced inflammatory response (López de Maturana et al., 2016). NLRP3-mediated release of proinflammatory cytokines IL-1β and IL-18 is thought to be important for neurodegeneration (Ramesh et al., 2013). Extensive increases in microglial activation and the NLRP3-related inflammasome have been observed in the SN of PD patients (Gordon et al., 2018). Furthermore, Sliter et al. (2018) reported a strong inflammatory phenotype in both Parkin^−/−^ and Pink1^−/−^ mice following exhaustive exercise and in Parkin^−/−^ mutator mice, which accumulate mutations in mtDNA. The authors reported that loss of STING, a central regulator of the type I IFN response to cytosolic DNA, rescued the loss of DA neurons from the SN and the motor defect observed in aged Parkin^−/−^ mutator mice (Sliter et al., 2018). These results support a role for PINK1 and Parkin in inhibiting innate immunity.

Glycogen synthase kinase 3β (GSK-3β) can phosphorylate downstream substrates, and it regulates glucose metabolism (MacAulay et al., 2007). One study has shown that the GSK-3β inhibitor effectively protects against 6-OHDA–induced neurotoxicity by blocking microglial activation in *in vitro* and *in vivo* models of PD (Morales-García et al., 2013). Thus, the production of proinflammatory factors such as TNF-α and IL-12 is repressed, and anti-inflammatory cytokines such as IL-10 are stimulated (Coant et al., 2011). Another study has shown that the decreased expression and activity of Na^+^-Ca^2+^ exchanger 3 (NCX3) in DA neurons may cause mitochondrial dysfunction and neuronal death in the midbrain of α-syn A53T mice, whereas NCX1 overexpression in microglia may promote their proliferation in the striatum (Sirabella et al., 2018). In addition, changes in metabolic signaling resulting from targeting mitochondrial pyruvate carrier are a key proprietor of cellular metabolism, which can regulate mTOR (mammalian target of rapamycin) activation, resulting in a neuroprotective and anti-inflammatory function in several PD models (Ghosh et al., 2016).

#### Mitochondria Targeted Therapy for PD

The development of mitochondrial targeting antioxidant seems to have made remarkable progress in the past decade. A nitrogenous guanidine compound called creatine has antioxidant properties and effectively regulates the opening status of MtPTPs; creatine is considered to be a neuroprotective agent (NINDS NET-PD Investigators, 2008). Coenzyme Q10 (CoQ10) has been identified to be another neuroprotective reagent in *in vitro* and *in vivo* PD models. Under oxidative stress, pretreatment with CoQ10 improves the mitochondrial membrane potential and decreases the production of ROS in neuronal cells (Xi et al., 2018). Coenzyme Q10 can also reduce oxidative damage and the behavioral abnormality in a rat model of PD (Gupta et al., 2018). Clinical trials show that a high dose of CoQ10 interferes with the aggravation of PD (Shults et al., 2002). In the 6-OHDA-induced PD model *in vivo* and *in* *vitro*, MitoQ, a mitochondria-targeted antioxidant, was found to improve the morphology and function of mitochondria by upregulating the level of Mfn2, which is the primary factor for promoting mitochondrial fusion (Xi et al., 2018). MitoQ treatment also reduces mitochondrial fragmentation, the production of ROS, and apoptosis (Xi et al., 2018). However, a double-blind clinical trial reported that there was no significant difference between MitoQ- and placebo-treated PD patients. This study also proposed that MitoQ was not appropriate for patients who have already lost parts of their DA neurons (Snow et al., 2010).

### Huntington’s Disease

Huntington’s disease is an autosomal dominant neurodegenerative inherited disorder, characterized by involuntary movements, cognitive decline, and neuropsychiatric changes. Huntington’s disease is caused by trinucleotide repeats of CAG (36 repeats or more) on the short arm of chromosome 4p16.3 in the Huntingtin (Htt) gene (MacDonald et al., 1993). With a PolyQ expansion at the N terminus, the mutant Huntington protein (mtHtt) has a toxic function causing neuronal death, particularly in the striatum and progressively in other parts of the brain (Ferrante et al., 1991; Andrews et al., 1999; Williams and Paulson, 2008). Mitochondrial dysfunction has been demonstrated to be strongly correlated with HD, and dysregulation of mitochondrial dynamics plays a key role in the development of HD (Johri et al., 2013; Zhao et al., 2019).

#### Abnormal Mitochondrial Dynamics and HD

Dynamin-related protein 1 hyperactivation and related mitochondrial fragmentation were observed in HD cell culture, animal models, and patient brains (Song et al., 2011; Reddy and Shirendeb, 2012; Guo et al., 2013). Transcriptional factor p53 was reported to be involved in mitochondria-mediated necrosis and fragmentation in HD, directly interacting with mitochondrial fission protein Drp1 (Guo et al., 2013, 2014). Research into HD knock-in mouse striatum cells has shown that MAPK can lead to Drp1 phosphorylation *in vitro* (Roe and Qi, 2018). Furthermore, Zhao et al. (2019) reported that oligomerization of ATAD3A, a mitochondrial protein interacting with Drp1, not only contributes to Drp1-mediated mitochondrial fragmentation, but also impairs mtDNA maintenance in HD. Notably, blocking the interaction between Drp1 and ATAD3A by the peptide—DA1—was able to correct mitochondrial fragmentation and mtDNA damage, as well as attenuating HD-associated neuropathology (Zhao et al., 2019). There is a possibility that altered mitochondrial trafficking contributes to neurodegeneration in HD (Schon and Przedborski, 2011). Huntingtin-associated protein 1 (HAP1) is one of the Htt-binding partners and is transported along the axons, and the p150Glued subunit of dynactin is an essential component of molecular motors. There are two main mechanisms that lead to dysfunction of neuronal transport in HD (Carmo et al., 2018). The first mechanism suggests that the altered Htt/HAP1/p150Glued complex is accompanied by a decrease of motor proteins associated with microtubules (Gauthier et al., 2004). Evidence reveals that mtHtt impairs mitochondrial axonal transport in human neurons *in vitro* and in neurons from transgenic mice (Trushina et al., 2004). Another mechanism recapitulated occurrence of steric inhibition of the microtubular flow as a result of the transfer of the motor proteins from the soluble pool to protofibrillar complexes in human HD-affected brains (Trushina et al., 2004). Disruption of axonal transport could lead to protein aggregation, which can result in the loss of neurotrophic support and evoke neuronal death, ultimately causing neuronal dysfunction (Gunawardena and Goldstein, 2005).

#### Abnormal Mitophagy and HD

PINK1 overexpression decreased the formation of morphologically abnormal mitochondria in *Drosophila* models of HD, demonstrating that abnormal mitophagy takes part in HD pathogenesis (Khalil et al., 2015). The presence of mtHtt results in neuronal damage by triggering defects in mitochondria, increasing the accumulation of dysfunctional mitochondria. Intihar et al. (2019) recapitulated that PGC-1α provides neuroprotective effects by activating autophagy and increasing the turnover of mtHtt aggregates (Intihar et al., 2019). Notably, intracellular aggregates of elongated Htt, a well-established autophagy substrate, were significantly decreased by memantine. Memantine also accelerated the clearance of damaged mitochondria (Hirano et al., 2019). Recently, some studies have focused on the inhibition or elimination of pathological mitophagy. Guo et al. (2016) showed that valosin-containing protein (VCP), as an mtHtt-binding protein, accumulated in mitochondria and caused excessive mitophagy by recruiting LC3 to the mitochondria *via* the LIR motif. Importantly, blocking VCP mitochondrial accumulation by disruption of the interaction between VCP and mtHtt reduced mitochondrial damage and attenuated HD-associated neuropathology.

#### Defective OXPHOS, Oxidative Stress, and HD

Mutant Htt protein accumulation may evoke OXPHOS damage by direct interaction with mitochondria, by transcriptional alterations, or by both mechanisms. It has been demonstrated that respiratory chain enzyme activities exhibit defects in complexes II, III, and IV in the striatum of HD patients (Gu et al., 1996). In addition, not only the expression of mtHtt but also the loss of Htt was shown to strongly influence mitochondrial transcription. PGC-1α is a transcriptional coactivator and is a master gene responsible for cellular energy metabolism (Zhang et al., 2019). There is a decrease in the level of PGC-1α in various HD models (McGill and Beal, 2006; Weydt et al., 2006; Chaturvedi et al., 2009; Johri et al., 2013), which results in its low level of transcription. Moreover, Intihar et al. (2019) speculated that there is a p53–HSF1–PGC-1α axis that integrates transcriptional dysregulation and mitochondrial dysfunction into one single pathway, although the underlying mechanism remains elusive (Intihar et al., 2019).

### Amyotrophic Lateral Sclerosis

Amyotrophic lateral sclerosis is a motor neuron (MN) disease (MND) characterized by progressive MN degeneration in the brain and spinal cord, leading to severe weakness and eventually paralysis of limb and trunk muscles (Taylor et al., 2016). In most cases, ALS arises in individuals without a family history of the disease (sporadic ALS), whereas in other cases ALS is inherited [familial ALS (fALS)]. There is a strong correlation between fALS and the mutant RNA processing protein, including Cu/Zn superoxide dismutase 1 (SOD1), TAR DNA-binding protein 43 (TDP-43), C9ORF72(C9), and fused in sarcoma (Ince et al., 2011; Taylor et al., 2016; Starr and Sattler, 2018; Genevini et al., 2019; Yamawaki et al., 2019). Pathological SOD1 and TDP-43 perturb multiple mitochondrial pathways, including mitochondrial dynamics, mitochondrial-related inflammation, and bioenergetics.

#### Abnormal Mitochondrial Dynamics and ALS

Point mutations in the gene that encodes SOD1 are one of the earliest discovered genetic causes related to fALS. There is an increment of glucose catabolic activity upstream of the mitochondrial aerobic respiration in SOD1G93A mice, indicating that mitochondrial dynamics represent the crucial site of the synaptic bioenergetic impairment in ALS (Ravera et al., 2019). Superoxide dismutase 1 mutations affect protein folding, which is a potential source of the toxicity that leads to mitochondria degeneration (Wright et al., 2019). Misfolded proteins aggregate within the intermembrane space of mitochondria, ultimately leading to membrane disruption (Salehi et al., 2015; Watanabe et al., 2016). The combined effect of these changes leads to defects in respiration, generation of free radicals, electron transport, and ATP synthesis (Magrané et al., 2012; Cenini et al., 2019). SOD1G93A is likely to promote mitochondrial fission and inhibit fusion activity (Wang et al., 2018b). Compared with WT SOD1 osteocytes, SOD1G93A osteocytes showed elevated levels of Drp1, resulting in mitochondrial fragmentation, and decreased levels of mitochondrial fusion protein OPA1. Furthermore, Mdivi-1, the putative mitochondrial fission inhibitor, reduced the amount of mitochondrial fragmentation and improved mitochondrial health in cells expressing SOD1G93A. Pickles et al. (2016) observed that several antibodies, specifically recognizing misfolded SOD1 protein in the SOD1G93A mice model, are correlated with mitochondrial damage. They found that SOD1 antibodies AMF7-63 and DSE2-3H1 detected fibrils in the spinal cord and that AMF7-63-reactive misfolded SOD1 is associated with swollen mitochondria (Pickles et al., 2016). In addition, Altman et al. (2019) showed that, compared with sympathetic neurons, cultured MNs displayed higher axonal and synaptic mitochondrial immobility. This finding suggests that the accumulation of mitochondria in the neuromuscular junction (NMJ) may play a significant role in MN function (Altman et al., 2019).

TDP-43 proteinopathy, which is caused by dysregulation or mutations in the TDP-43 gene, can cause a series of neurodegenerative diseases such as ALS and frontotemporal lobar degeneration. Mutant TDP-43 causes fALS, which accounts for approximately 4% of fALS cases (Taylor et al., 2016). Although the mechanisms of mitochondrial dynamics regulated by TDP-43 remain unclear, TDP-43 appears to have a significant impact on mitochondrial fission and fusion. Xu et al. (2010) were the first to show that overexpression of WT TDP-43 induced abnormal juxtanuclear aggregates of mitochondria and increased the levels of Fis1 and phosphorylated Drp1, key components of the mitochondrial fission machinery, as well as decreasing mitofusin 1 expression, an essential component of mitochondrial fusion. Moreover, excessive mitochondria fission, together with the loss of mitochondrial inner membrane structure, occurs in neuron cells expressing ALS-associated mutant TDP-43 (Wang et al., 2013; Magrané et al., 2014; Gautam et al., 2019a). TDP-43 can interact with Mfn2, and TDP-43 selectively expressed by the cortex and hippocampus of humans can induce an age-dependent change in Mfn2 expression (Wang et al., 2013). While knocking down TDP-43 in HEK293T cells resulted in a notable reduction in Mfn2, overexpression of TDP-43 increased the Mfn2 levels (Davis et al., 2018).

TAR DNA-binding protein 43 plays a critical role in mediating mitochondrial transport. Both overexpression of WT TDP-43 and knockdown of TDP-43 can cause dysregulation of mitochondrial anterograde and retrograde transport in cultured primary MNs (Wang et al., 2013). These findings have also been observed in fly and mice models, which further confirm the function of TDP-43 in mitochondrial transport (Magrané et al., 2014; Baldwin et al., 2016). Interestingly, the speed of mitochondria transport appeared to be slower in human induced pluripotent stem cells-derived MNs with TDP-43 mutation. Nevertheless, no detectable cytoplasmic inclusions or phosphorylated TDP-43 aggregation was observed in these neurons, which indicates that mutant TDP-43 can give rise to mitochondrial toxicity and is not associated with proteinopathy (Kreiter et al., 2018). Moreover, the cytoskeleton plays a vital role in the intracellular transport and localization of mitochondria (Chetta et al., 2015); cytoskeleton deficits that result from pathological TDP-43 may enhance mitochondria dysregulation (Oberstadt et al., 2018).

#### Aberrant Mitophagy and ALS

Utilizing a proteomics screen, Davis et al. (2018) identified several mitochondrial proteins that interact with TDP43 in a mouse model of MND, such as voltage-gated anion channel 1 and prohibitin 2 (PHB2), a key mitophagy receptor. Overexpression of TDP-43 induced elevated levels of PHB2, whereas TDP-43 knockdown decreased PHB2 expression in cells exposed to carbonyl cyanide m-chlorophenylhydrazone, an inducer of mitophagy (Davis et al., 2018). Recently, Gautam et al. (2019b) discovered a novel pathway of mitochondrial clearance before neuronal vulnerability, called mitoautophagy. They proposed that mitochondria can clear themselves independently of autophagosome-mediated degradation, which is different from mitophagy. Furthermore, they highlighted that mitoautophagy mainly presents in the upper MNs of PFN1G118V and prpTDP-43A315T mice, similar to many characteristics of the disease in patients with TDP-43 pathology. In addition to TDP43, the mutant SOD1 suppresses the axonal transport of mitochondria by promoting PINK1/Parkin-dependent Miro1 degradation (Moller et al., 2017).

#### Defective OXPHOS, Oxidative Stress, and ALS

Metabolic changes and stress responses occur in the lumbar spinal cord of SOD1G93A mutant mice prior to the onset of motor symptoms (Pharaoh et al., 2019). In SOD1G93A mice, there is only a decrease at the last step of the respiratory chain (complex IV), which disrupts the association of cytochrome *c* with the IMM, thereby eliciting the apoptotic program (Kirkinezos et al., 2005). Maekawa et al. (2014) reported that oxidative stress and autophagic alteration take place not only in the brain and spinal cord of SOD1G93A mice but also in the brainstem of these mice. Several studies have shown that mitochondrial OXPHOS deficits occur in experimental models associated with TDP-43. Full-length TDP-43 within mitochondria impairs the assembly and function of subunits (ND3/6) of the OXPHOS complex I, which is encoded by mitochondria-transcribed mRNAs. However, truncated TDP-43 had no effect on OXPHOS complex I (Wang et al., 2016, 2019; Salvatori et al., 2018). In a similar study, by electron microscopy analysis, mitochondrial damage in both cellular and animal models of TDP-43 proteinopathy including abnormal cristae, as well as loss of cristae, has been observed. In these models, overexpression of TDP-43 triggered mitochondrial dysfunction, including reduced mitochondrial membrane potential and increased ROS production (Onesto et al., 2016; Bartolome et al., 2017; Wang et al., 2019). Exogenous-added TDP-43 was demonstrated to aggregate inside neuronal cells, triggering ROS generation (Capitini et al., 2014). Moreover, TDP-43 expression inhibited mitochondrial complex I activity and subsequently suppressed mitochondrial ATP synthesis (Wang et al., 2019). Similarly, mitochondrial complexes II and IV are also shown to be dysregulated in ALS (Tabassum and Jeong, 2019). Bioenergetic deficits may bring a fatal effect on mitochondria themselves. Dang et al. (2020) demonstrated that the binding of decoded ATP improves thermodynamic stability of TDP-43 RRM (RNA-recognition motif) domains, followed by inhibition of ALS-associated fibrillation. This suggests that the decrease of ATP would promote fibrillation in the neurons of ALS patients (Dang et al., 2020).

#### Mitochondrial-Driven Inflammation and ALS

There is evidence of spinal cord infiltration by macrophages and T cells, and it was demonstrated that some MNs were ingested by IL-6- and TNF-α-positive macrophages in postmortem ALS spinal cords (Lam et al., 2016). Although the mechanism of mitochondrial-driven inflammation in ALS is still elusive, there is evidence that SOD-1 is critically correlated with inflammation. For example, elevated levels of cytokine macrophage migration inhibitory factor could enhance neuronal survival by blocking the accumulation of misfolded SOD-1 in mitochondria (Israelson et al., 2015). Moreover, misfolded SOD-1 can be detected by several conformationally restricted antibodies, such as AMF7-63. AMF7-63 recognizes the spinal cord mitochondria with misfolded SOD1, in which volume homeostasis dysregulation and superoxide generation are enhanced (Pickles et al., 2016). In MNs, mutations in the TDP43 gene can initiate a neuroinflammatory response causing further neuronal damage. Joshi et al. (2019) reported that inflammation propagation in ALS is greatly triggered by the release of dysfunctional and fragmented microglial mitochondria into the neuronal environment, and subsequent neuronal damage depends on Fis1-mediated mitochondrial disruption in glial cells.

#### Mitochondria Targeted Therapy for ALS

Several lines of evidence show that the protection of mitochondrial activity could be protective in ALS rodent models. Dexpramipexole is a dopamine agonist that scavenges ROS in order to improve metabolic efficiency. Treatment with dexpramipexole has been shown to reduce motor deficits and improve the survival of mutant SOD1G93A mice (Danzeisen et al., 2006). In a phase II clinical trial, dexpramipexole was shown to be effective in a dose-dependent manner for ALS patients (Cudkowicz et al., 2011). However, there was no significant difference between placebo and dexpramipexole in the subsequent phase III clinical trial (Cudkowicz et al., 2013). Trehalose, an autophagic inducer, can prolong the survival of SOD1G93A mice by protecting mitochondria and decreasing SOD1 and SQSTM1/p62 aggregation (Zhang et al., 2014). Olesoxime directly binds to proteins of the OMM and acts on the MPTP. Olesoxime can prevent MN death, activate microglia and prolong survival of SOD1G93A mice (Sunyach et al., 2012; Yang et al., 2013). However, in a phase II–III trial, the survival time of patients did not appear to have an obvious difference between placebo- and olesoxime-treated cohorts. Thus, whether targeting mitochondria could provide a translational strategy for treating ALS patients remains to be elucidated and should be investigated further.

## Concluding Remarks and Future Perspectives

Neurodegenerative diseases, including AD, PD, HD, and ALS, are all characterized by progressive neuronal degeneration in specific brain regions. To date, therapeutic strategies are ineffective for these neurodegenerative diseases. Mitochondria regulate cellular metabolism, coordinate cell death, play a role in viability pathways, and are essential for maintaining neuronal integrity. In the past decade, accumulating evidence has demonstrated that mitochondrial dynamics and mitophagy serve as common and initial characteristics that account for mitochondrial and neuronal dysfunction in these diseases. Notably, manipulation of mitochondrial dynamics and mitophagy have been shown to be protective in these diseases, which provides new therapeutic options toward developing effective treatments.

Furthermore, increasing evidence suggests that mitochondria could be a hub for regulating innate and adaptive immune responses. Neuroinflammation occurs in experimental animal models and patient brains of several neurodegenerative diseases. However, the role of neuroinflammation is still controversial and could either be the dominant pathogenesis of neuronal damage or only a result of metabolic dysfunction in the neurodegenerative progress. Future investigations on the cellular and molecular crosstalk between mitochondrial dysregulation and immune responses, along with their contribution to disease progression, may deepen our understanding of the interaction between neuroinflammation and neurodegeneration.

## Author Contributions

XY and XZ made substantial contributions to the conception and design of the study. XY, BW, YH, SW and XZ participated in drafting the article. XY and XZ gave final approval of the version to be submitted and revised versions.

## Conflict of Interest

The authors declare that the research was conducted in the absence of any commercial or financial relationships that could be construed as a potential conflict of interest.
